# The effect of mindfulness-based stress reduction on anxiety and sleep quality in informal family caregivers of cancer patients: a randomized controlled trial

**DOI:** 10.1186/s12912-025-04063-z

**Published:** 2025-11-06

**Authors:** Sayedeh Farkhondeh Safavi, Hakimeh Vahedparast, Batool Amiri, Taiebeh Gharibi, Azam Hosseinnejad

**Affiliations:** 1 M.Sc. Student of Nursing, Student Research Committee, School of Nursing and Midwifery, Bushehr University of Medical Sciences, Bushehr, Iran; 2 Department of Medical-Surgical Nursing, School of Nursing and Midwifery, Bushehr University of Medical Sciences, Bushehr, Iran; 3 Clinical Research Development Center, Bushehr University of Medical Sciences, Bushehr, Iran; 4 Department of Midwifery, School of Nursing and Midwifery, Bushehr University of Medical Sciences, Bushehr, Iran

**Keywords:** Mindfulness-based stress reduction, Anxiety, Sleep quality, Family caregivers, Cancer

## Abstract

**Background:**

Informal caregivers play a crucial supportive role throughout the course of illnesses, particularly in patients with cancer. Following a cancer diagnosis, informal caregivers often experience substantial psychological, physical, and social stress, which may lead to conditions such as anxiety and sleep disturbances. Therefore, implementing interventions aimed at reducing anxiety and improving sleep quality is essential. The present study aims to assess the impact of mindfulness-based stress reduction (MBSR) training on anxiety and sleep quality among informal caregivers of patients with cancer.

**Methods:**

This randomized controlled trial was conducted in 2024 on 66 informal caregivers of cancer patients in Bushehr, Iran. Participants were selected using convenience sampling method and then randomly assigned to either the intervention or control group. The intervention group received eight sessions of MBSR training, each lasting 120 min, which included mindfulness meditation, body scan, and mindful breathing exercises. The control group received no intervention. Data were collected using a demographic form, Spielberger State-Trait Anxiety Inventory (STAI), and Pittsburgh Sleep Quality Index (PSQI). Data were analyzed using SPSS 26.0 through descriptive statistics, independent t-test, paired t-test, and ANCOVA at a significance level of 0.05.

**Results:**

The results indicated a statistically significant difference between the two groups regarding both anxiety and sleep quality after the intervention (anxiety: *P* < 0.001, ηp² = 0.22; sleep quality: *P* = 0.004, ηp² = 0.12). In the intervention group, the mean anxiety score decreased from 105.94 ± 11.73 (pretest) to 80.39 ± 13.68 (one-month follow-up), and the mean sleep quality score (PSQI) improved from 11.26 ± 2.65 to 9.94 ± 2.09. In contrast, the control group showed no significant improvement.

**Conclusion:**

The results of this study suggest that mindfulness-based interventions may help reduce anxiety and improve sleep quality in female informal caregivers of cancer patients. This intervention shows promise as a potentially effective approach for promoting caregivers’ mental health. However, the findings are only applicable to women and cannot be generalized to male caregivers, who may experience different social and emotional burdens. In addition, the use of a passive control group and the dual role of the instructor (intervention delivery and homework monitoring) represent important limitations. Furthermore, due to the small sample size, use of convenience sampling, and partial blinding, the results should be interpreted with caution. Future research should include both genders, use active control conditions, and conduct larger long-term randomized trials to confirm these outcomes and assess their generalizability across different settings.

**Trial registration:**

IRCT, IRCT20240310061244N1, registered prospectively on 2024-04-30.

**Supplementary Information:**

The online version contains supplementary material available at 10.1186/s12912-025-04063-z.

## Background

**Informal caregivers**, often referred to as the hidden workforce, play an essential role in supporting cancer patients by providing both emotional and direct care [1].Cancer is increasingly conceptualized as a “we-disease,” emphasizing its profound impact not only on patients but also on their families and close relatives. These caregivers assist with medical visits, treatment management, medication adherence, and financial matters, all while managing their own daily responsibilities [1, 2].They are instrumental in facilitating patients’ adaptation to illness and adherence to treatment protocols [3].

According to the GLOBOCAN 2022 report, approximately 19.3 million new cancer cases were reported worldwide [4], and more than 6 million informal caregivers are now engaged globally [5]. In Iran, cancer incidence is also on the rise; the World Health Organization reported nearly 140,000 new cancer cases in 2020 [6]. This trend reflects a growing demand for informal caregiving within the Iranian healthcare context. Although they constitute only 7–15% of the total caregiver population, informal cancer caregivers face unique challenges due to the acute onset and unpredictable course of cancer compared to other chronic illnesses like dementia [7].

Psychological distress is prevalent among these caregivers, with studies reporting high rates of stress, anxiety, depression, and sleep disturbances [8–11]. One major contributor to these outcomes is cognitive hyperarousal, a psychological state characterized by persistent mental alertness and intrusive worry about the patient’s prognosis, treatment course, and financial implications [12]. This hyperarousal interferes with sleep initiation and maintenance, thereby aggravating anxiety and creating a bidirectional cycle of psychological distress and poor sleep quality [13].

Cognitive hyperarousal is considered a core mechanism in chronic insomnia, and research suggests that metacognitive beliefs**—**individuals’ beliefs about the significance, controllability, or danger of their own thoughts—may sustain this state. Challenging such beliefs may reduce anxiety and improve sleep; however, direct empirical research in caregiver populations remains limited, particularly in non-Western contexts [14].

Mindfulness-Based Stress Reduction (MBSR) is a structured, complementary approach that integrates mindfulness meditation and gentle Hatha yoga to cultivate nonjudgmental awareness of present-moment experiences. It is believed to enhance metacognitive awareness, emotion regulation, and attentional control [15]. Previous studies have shown that MBSR can improve anxiety, sleep quality, and overall psychological well-being in informal caregivers [16–19], though findings remain inconsistent due to variations in methodology, intervention delivery, and participant populations [20, 21].

The effectiveness of mindfulness interventions is shaped by cultural context. In Iran, cultural values such as familial obligation, collectivism, religious coping, and spiritual resilience strongly influence caregiving roles and emotional responses to illness. For example, caregiving is often perceived as a religious or moral duty, which may heighten psychological pressure due to internalized expectations These factors may either enhance or hinder the acceptability and impact of mindfulness interventions. However, few studies have examined how such interventions function within Iranian cultural frameworks, representing a significant research gap.

Moreover, nurses play a critical role in promoting the psychological, physical, and social empowerment of caregivers. As frontline healthcare professionals, nurses often build trusting relationships with both patients and their families [22, 23]. Holistic care models and nursing ethics advocate the integration of complementary therapies, such as mindfulness, to address caregiver burden comprehensively [24].

Therefore, this study aims to examine the effectiveness of mindfulness training on anxiety and sleep quality among informal caregivers of cancer patients in Bushehr, Iran, while considering the cultural, familial, and professional caregiving context.

## Methods

### Study design and setting

This study was a randomized controlled trial (RCT) with a pretest, posttest, and one-month follow-up design, including intervention and control groups. Participants were recruited from the hematology department of Shohadaye Khalij-e Fars Hospital—the main referral and central hospital of Bushehr Province—and from oncology clinics in Bushehr, a coastal city in southern Iran along the Persian Gulf. Eligible participants were randomly allocated to either the intervention or control group using a block randomization method to ensure group balance. A total of 11 blocks were used, consisting of 3 blocks of size 4, 3 blocks of size 6, and 5 blocks of size 8, generated through a computer-based randomization program. Allocation concealment was maintained through the use of sealed, opaque, and sequentially numbered envelopes, which were prepared by an independent researcher not involved in participant recruitment or intervention delivery. Due to the nature of the intervention, blinding of participants and instructors was not feasible. However, to minimize potential bias, the outcome assessors and data analysts were blinded to group allocation throughout data collection and analysis.

### Participants

Informal caregivers, defined as unpaid individuals—primarily family members—who assist cancer patients with daily living activities, serve as the first line of support by providing emotional care, managing household responsibilities, communicating with healthcare teams, and offering financial or logistical assistance [1], In this study, individuals were identified as primary caregivers if they (1) were the main person responsible for providing daily care to the patient, and (2) reported spending the most time with the patient during the day. This identification was based on self-report during the initial screening interview and was applied consistently by trained research assistants. Participants were recruited using a convenience sampling method, as described above.

Inclusion criteria included being female, aged 18–65 years, providing informed consent, caring for patients aged 18 or older, being literate in Persian, having at least three months elapsed since the patient’s last major treatment (chemotherapy, radiotherapy, or surgery) to ensure post-acute stabilization and minimize variability related to acute treatment effects [25], acknowledgment by both patient and participant of the caregiver’s primary role, and scoring ≥ 32 on the Spielberger State-Trait Anxiety Inventory (STAI), a widely validated tool with scores ranging from 20 to 80, where 32 indicates moderate or higher levels of anxiety [26, 27].

Exclusion criteria included caregiving during the patient’s acute treatment phase; diagnosed psychiatric disorders; ongoing substance abuse; bereavement due to the death of a close relative in the past year; participation in concurrent psychosocial interventions; regular engagement in mindfulness-related practices (e.g., meditation or yoga); development of a health condition that would interfere with program attendance; and clinical deterioration or death of the patient during the intervention. In addition, only clinically verified sleep-related conditions were considered exclusionary (e.g., diagnosed insomnia or use of prescribed sleep medications), in order to avoid ambiguity associated with self-reported sleep complaints.

### Sample size

**Fig. 1:**
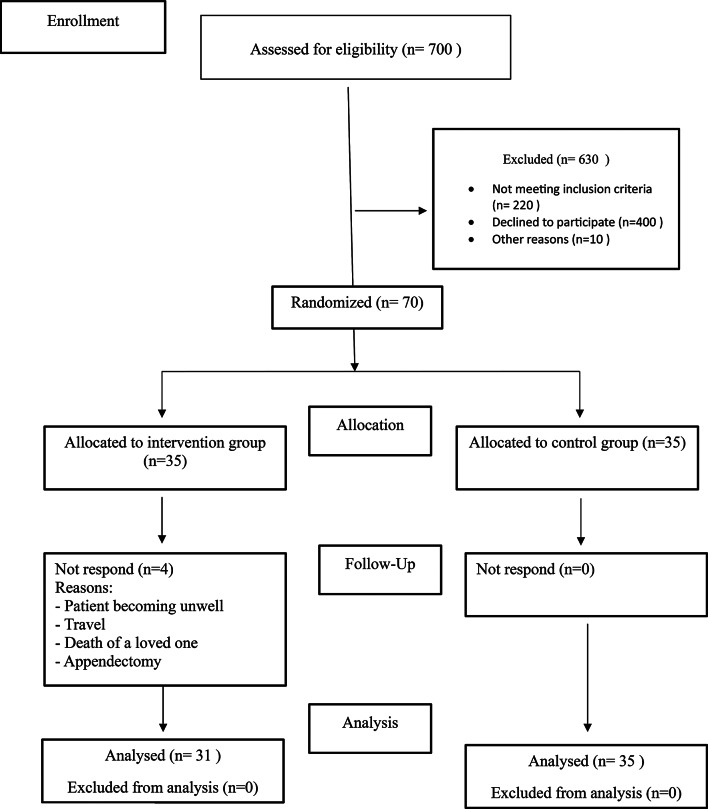
The consort diagram of the trial

The required sample size was estimated using G*Power v.3.1.9.4, based on a repeated-measures ANOVA with two groups (intervention and control), three time points (pretest, posttest, and one-month follow-up), and the interaction effect of group × time as the main parameter of interest. Assuming an alpha level of 0.05, power (1-β) of 0.80, a small-to-medium effect size (Cohen’s f = 0.15; equivalent to d ≈ 0.3), and a correlation among repeated measures of 0.5, the estimated sample size was 31 participants per group (62 in total). To account for potential attrition, a 10% dropout rate was anticipated, leading to a final target sample size of 70 participants. A total of 700 informal caregivers were screened over a four-month period in hematology and oncology units in Bushehr. Of these, 220 did not meet the inclusion criteria, 400 declined participations, and 10 were excluded due to incomplete assessments. Ultimately, 70 caregivers were enrolled and randomly assigned to the two groups (Fig. 1).

During the intervention, 4 participants in the intervention group discontinued participation due to personal reasons, including: patient health deterioration, travel, loss of a loved one, and undergoing an appendectomy. Thus, 66 participants (*n* = 31 intervention, *n* = 35 control) completed all three phases of the study and were included in the final analysis.

Although formal documentation of refusal reasons was not obtained, informal feedback suggested that time constraints, summer heat, and logistical challenges played a major role in non-participation. These factors may have contributed to the relatively high refusal rate, potentially introducing selection bias.

### Data collection tools

Data were collected using a demographic form and two standardized questionnaires. The demographic form separately gathered information about caregivers and patients to avoid ambiguity: caregiver variables included age, marital status, educational level, employment status, income, and duration of caregiving; patient variables included type of cancer and presence of another caregiver. These variables were clearly identified for separate analyses.

All questionnaires were administered in person by trained research assistants, using paper-based self-report forms at three time points: baseline (pre-intervention), immediately post-intervention, and one-month follow-up. The research assistants were blinded to group allocation to reduce assessment bias.

The 19-item Pittsburgh sleep quality index (PSQI) was used to assess sleep quality. This scale consists of 7 subscales, including subjective sleep quality, sleep latency, sleep duration, sleep efficiency, sleep disturbances, use of sleeping medications and daytime dysfunction. Subjective sleep quality is assessed using question 6; sleep latency is measured by questions 2 and 5a; sleep duration by question 4; sleep efficiency by questions 1, 3, and 4; sleep disturbances by questions 5b through 5j; use of sleeping medications by question 7; and daytime dysfunction by questions 8 and 9. The total score of this questionnaire ranges from 0 to 21, with a score higher than 5 indicating poor sleep quality. Each item is scored on a 4-point Likert scale from 0 to 3 [28]. The Persian version validated by Toroghi et al. (2020) was used, which demonstrated a validity coefficient of 0.86 and reliability of 0.89 [29]. In the present study, reliability was calculated, and the Cronbach’s alpha coefficient was 0.80.

The Spielberger State-Trait Anxiety Inventory (STAI) was used to assess anxiety. This questionnaire consists of 40 items divided into two distinct subscales: 20 items measure state anxiety, and 20 items measure trait anxiety. Each subscale yields a score ranging from 20 to 80, with higher scores indicating greater anxiety. Items are rated on a 4-point Likert scale, ranging from 1 (not at all) to 4 (very much so) [26]. The Persian version validated by Gholami Bourang et al. (2017) was used, showing reliability of 0.87 and convergent validity of 0.65 [27]. In the present study, reliability was calculated, and the Cronbach’s alpha coefficient was 0.95. Results were analyzed based on the mean total anxiety score across all 40 items (state and trait combined), in accordance with the study objectives. While the STAI consists of two distinct subscales, in this study we used the overall mean score as a general indicator of caregiver anxiety.

### Data collection

The study commenced after obtaining approval from the Research Ethics Committee of Bushehr University of Medical Sciences (IR.BPUMS.REC.1403.011) and receiving a formal letter of introduction from the Vice Chancellor for Research. Necessary coordination and authorization were also obtained from the managers of participating clinical centers.

Eligible patients were identified through review of medical records. To determine their primary informal caregiver, patients were asked to identify the individual who provided the majority of their unpaid, day-to-day physical and emotional support. This approach aligns with the definition provided by the World Health Organization and has been used in similar caregiver studies. Patients were either contacted directly or informed during clinic visits.

Once the primary caregiver was identified, they were approached for participation. Trained research staff, separate from the intervention team, provided a full explanation of the study, including its voluntary nature, intervention procedures, and data confidentiality. After informed consent was obtained, caregivers completed the State-Trait Anxiety Inventory (STAI) to determine eligibility. Caregivers with a baseline state anxiety score of ≥ 32 were included in the study.

Participants were then randomly assigned to the intervention or control group (35 participants each) using a block randomization method. To ensure allocation concealment, a sequence was generated using the Sealed Envelope website, and assignments were placed inside sealed, opaque, sequentially numbered envelopes by an independent coordinator. The envelope for each participant was opened only after baseline assessments were completed.

At baseline, participants completed the demographic questionnaire, the Pittsburgh Sleep Quality Index (PSQI), and the STAI. To minimize performance and detection bias, the intervention was delivered by a facilitator not involved in recruitment or assessment. Additionally, data collection at all time points was conducted by blinded research assistants who were unaware of group assignment. The data analyst was also blinded during the analysis phase.

The study was conducted from April to July 2024 and consisted of three clearly defined phases:

- Phase 1 (Pre-intervention): Baseline data collection (demographic form, PSQI, STAI).
- Phase 2 (Intervention): Eight weekly MBSR sessions for the intervention group; the control group received no treatment. Immediately after the final session, both groups completed the PSQI and STAI again.
- Phase 3 (Follow-up): One month after the final session, both groups completed the PSQI and STAI for the third time.

Four participants in the intervention group discontinued participation for non-intervention-related reasons, including illness, travel, bereavement, and surgery. However, all randomized participants were retained in the final analysis based on the intention-to-treat (ITT) principle. Missing outcome data were addressed using the Last Observation Carried Forward (LOCF) method. No further attrition occurred after the initial withdrawals, and follow-up data completion among remaining participants was 100%.

### Intervention

The intervention group participated in eight weekly sessions of Mindfulness-Based Stress Reduction (MBSR), each lasting 120 min, over a two-month period. The program was implemented following the Kabat-Zinn protocol [30], using a Persian-adapted version previously validated in Iranian populations [25] to ensure cultural relevance and acceptability. The instructor, holding a master’s degree in clinical psychology and certified in mindfulness, had extensive experience in conducting mindfulness courses. Prior to the intervention, the most convenient session times were determined based on participants’ preferences, and detailed schedules were communicated via phone calls. Sessions were held in a calm, quiet environment equipped with audiovisual facilities, located to ensure easy access for participants.

To enhance the effectiveness of the training, the intervention group was divided into two smaller groups (17 and 18 participants respectively), as smaller group sizes facilitate more effective mindfulness training. Each session covered structured content aligned with the Kabat-Zinn protocol (summarized briefly here: introduction to mindfulness concepts, body scan, mindful breathing, yoga and gentle movement, dealing with stress and emotions, and applying mindfulness in daily life; see Table 1 for full details).

To reinforce learning, participants received weekly homework assignments, guided audio files, and notebooks to record their experiences. The instructor maintained weekly follow-ups via WhatsApp and phone to encourage adherence.

To preserve continuity, compensatory sessions were provided in-person or via a structured educational package, which included video recordings of missed content, guided audio practices, and a printed session summary. These materials were reviewed for fidelity by the research team to ensure equivalence.

Protocol fidelity was monitored through instructor checklists, session attendance logs, and periodic supervision meetings with a clinical psychologist unaffiliated with the intervention delivery.

The checklist covered key elements such as adherence to session content (e.g., body scan, mindful breathing), time management, and participant engagement. Supervision meetings addressed delivery consistency, cultural adaptations, and logistical challenges.

To minimize bias, the instructor was not involved in outcome assessment or statistical analysis. However, due to resource constraints, an attention-control or placebo condition was not employed for the control group. The control group received no intervention during the study period to avoid contamination of data. However, for ethical reasons, they were provided with an MBSR CD and printed materials after the study was completed.

**Table 1:** The description of the MBSR sessions

Session	Topics	Assignments
First session	Introduction of participants and intervention, the raisin eating technique, body scan meditation, and assignment presentation	Relaxing breathing exercise
Second session	Body scan, distinguishing between thoughts and feelings, sitting meditation	Encourage sitting meditation (10–15 min), recording pleasant experiences
Third session	Observing Exercise and Focusing on Emotions	sitting meditation with Abdominal Breathing (15–20 min) Before Sleep, Completing the Daily Pleasant Events Calendar, and Recording Daily Emotions
Fourth session	Meditation with Breath Awareness and the Exercise of Expressing and Listening to Emotions	Body Scan with Yoga (45 min Daily) and Recording Unpleasant Daily Events
Fifth session	Meditation, Self and Emotional Acceptance	Starting Walking Meditation and Noticing Physical Sensations, Sounds, Thoughts, and More
Sixth session	Imagination, Non-Judgment of Emotions and Events	Daily sitting Meditation (30–45 min) Alternating with Yoga and Using Breath as a Tool for Attention Control
Seventh session	Meditation and Awareness of the Present Moment	Mindful Eating and Body Scan with Yoga (30–40 min)
Eighth session	Reviewing Experiences and Conducting a Post-Test	Course Review & Emphasizing Daily and Consistent Practice of Educational Exercises

### Statistical analysis

Data collected via coded questionnaires were entered into SPSS version 26.0 and analyzed using descriptive and inferential statistical methods. Descriptive statistics included means and standard deviations for quantitative variables, and frequencies and percentages for qualitative variables. Baseline demographic variables were compared between the intervention and control groups using independent samples t-test, Chi-squared test, Fisher’s exact test, and Chi-squared test for trend.

For comparing mean scores between groups, independent samples t-tests were employed. To evaluate changes in mean scores over time between the two groups, repeated measures analysis of covariance (ANCOVA) was applied. In the ANCOVA model, Duration of care was included as covariates to control for potential confounding. An unstructured covariance matrix was used to model within-subject correlations in the repeated measures data. Within-group comparisons were conducted using paired samples t-tests.

To assess the normality of study variables and determine the suitability of parametric tests, the Shapiro-Wilk test was applied, complemented by visual inspection of quantile-quantile (Q-Q) plots. In cases where normality assumptions were violated, non-parametric alternatives such as the Mann-Whitney U test and Friedman test were used.

To address missing data, multiple imputation was performed under the assumption of missing at random (MAR), creating multiple imputed datasets. Both intention-to-treat (ITT) and per-protocol (PP) analyses were conducted, and results from both approaches are reported to provide a comprehensive evaluation of the intervention effects.

Where multiple comparisons were made, the Bonferroni correction was applied to control for Type I error inflation. A significance level of *p* < 0.05 was considered for all statistical tests.

## Results

The results showed that the mean age of caregivers in the intervention and control groups was 39.81 ± 9.82 and 42.09 ± 10.91 years, respectively. As shown in Table 2 and Additional files 1 and 2, the intervention and control groups were comparable at baseline on most demographic and clinical variables, including caregiver age, gender, marital status, education level, employment status, patient-caregiver relationship, duration of caregiving, and patient characteristics such as cancer type and presence of another caregiver. However, a statistically significant difference was observed in caregiving duration (*p* = 0.025), assessed using the Mann–Whitney U test. This variable was therefore included as a covariate in the final repeated measures ANCOVA model. Most caregivers were female (68.2% in the intervention group, 65.7% in the control group).

As presented in Table 3, the intervention had a statistically significant effect on both anxiety and sleep quality. For anxiety, the repeated measures ANCOVA yielded F (2, 126) = 9.43, *p* < 0.001, partial eta squared (ηp²) = 0.22, indicating a large effect size based on Cohen’s guidelines. For sleep quality, F (2, 126) = 4.67, *p* = 0.011, ηp² = 0.12, indicating a moderate effect.

**Fig. 2:**
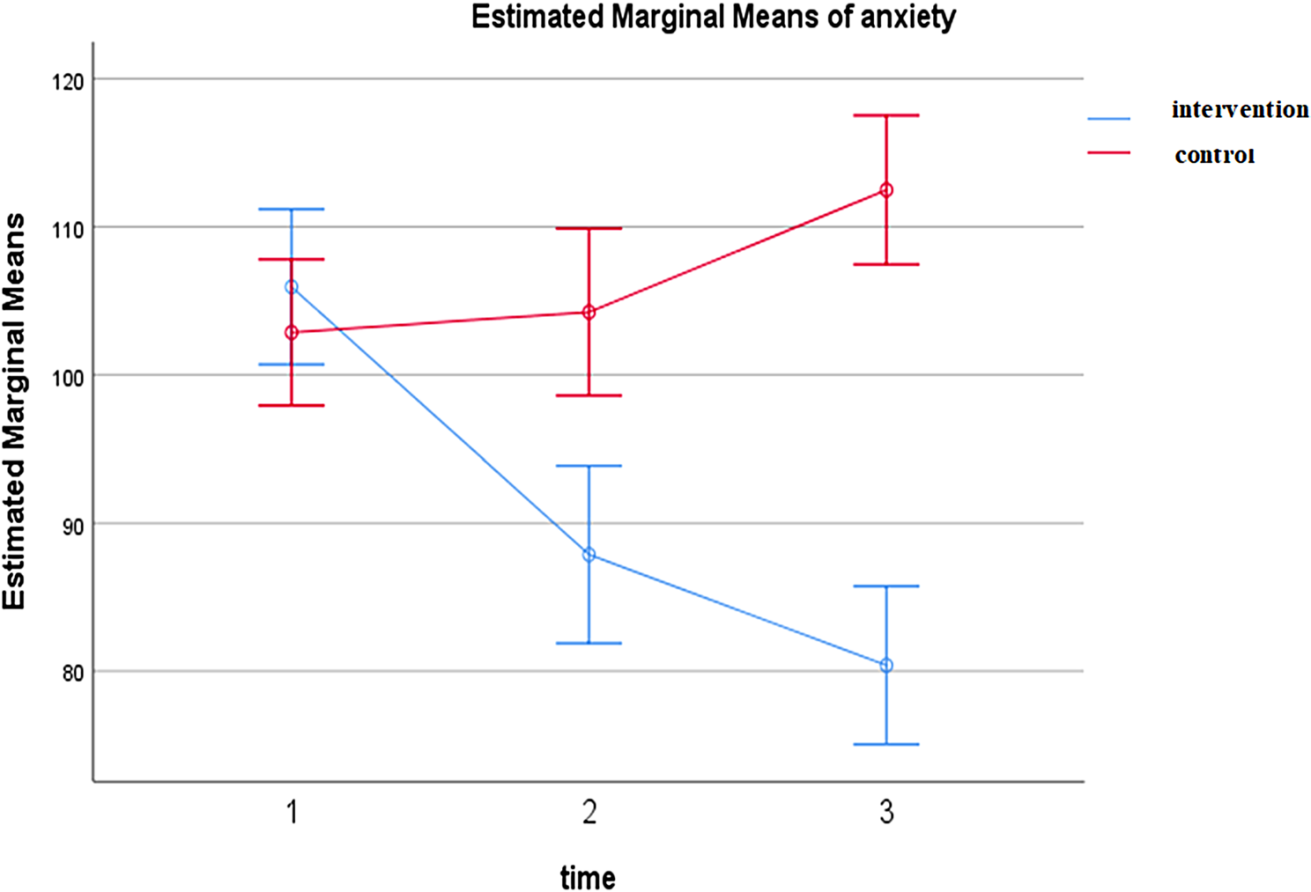
Comparison of changes in the mean anxiety score between the intervention and control groups

**Fig. 3:**
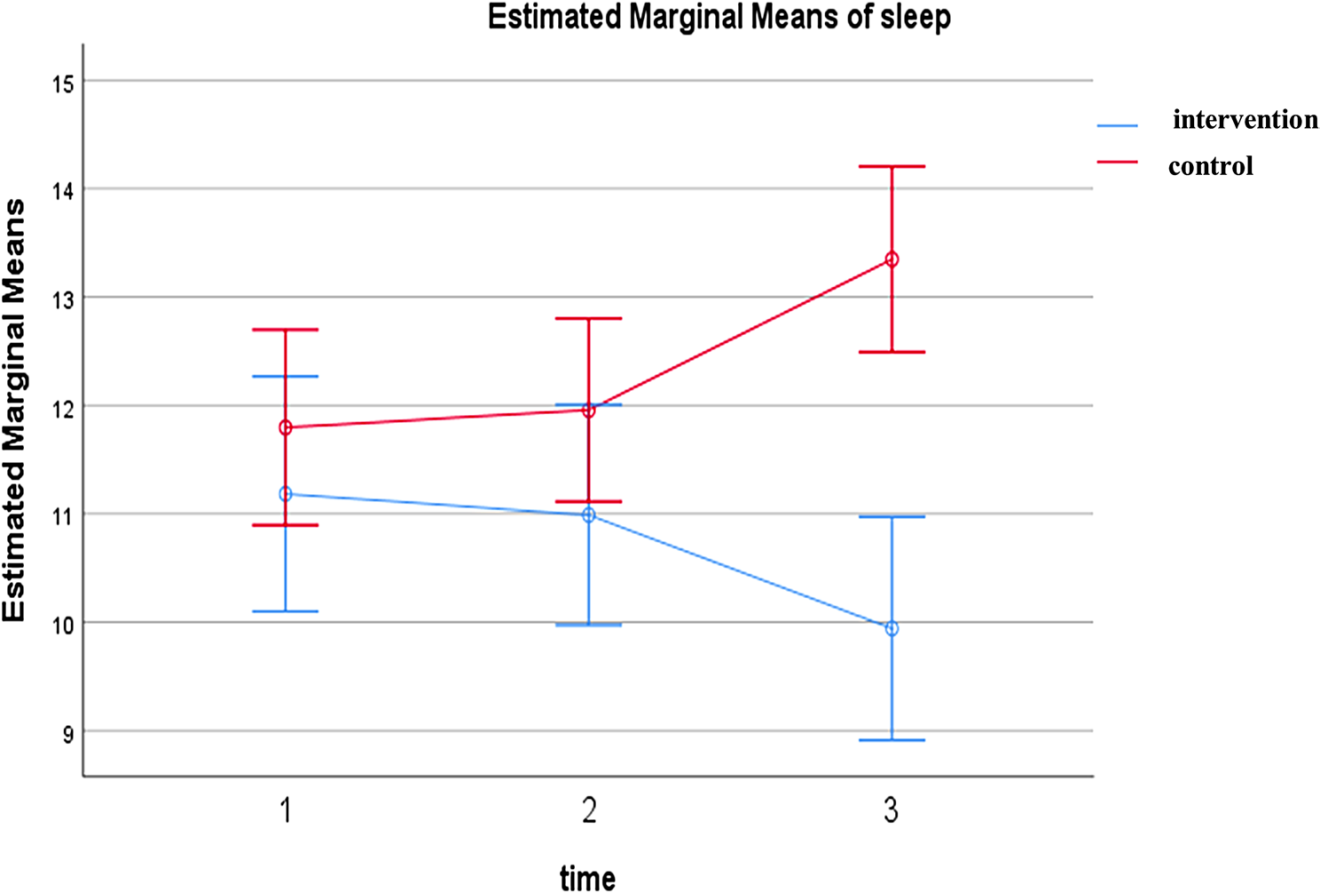
Comparison of changes in the mean sleep quality score between the intervention and control groups

Figures 2 and 3 display the trends over time: Fig. 2 shows a consistent decrease in anxiety scores in the intervention group compared to relative stability in the control group; Fig. 3 shows improvement in sleep quality over time in the intervention group compared to minimal change in the control group.

Additionally, the improvements in both outcomes were sustained at the one-month follow-up (T3), with significant between-group differences still present.

**Table 2:** Comparison of demographic variables of caregivers between intervention and control groups

Variable	Intervention group frequency (%)	Control group frequency (%)	Fisher or x2	*P* value
Marital status				
Single Married Divorced Widow	8 (25.8) 20 (64.5) 2 (6.5) 1 (3.2)	4 (11.4) 27 (77.1) 2 (5.7) 2 (5.7)	2.62^**^	0.466
Education				
Under diploma Diploma Associate Bachelor Master	5 (16.1) 10 (32.3) 4 (12.9) 11 (35.5) 1 (3.2)	9 (25.7) 11 (31.4) 6 (17.1) 8 (22.9) 1 (2.9)	2.08^**^	0.786
Job status				
Housewife Employee Self-Employed Unemployed	18 (58.1) 8 (25.8) 5 (16.1) 0 (0)	24 (68.6) 6 (17.1) 3 (8.6) 2 (5.7)	3.05^**^	0.408
Income				
Good Average Poor	2 (6.5) 18 (58.1) 11 (35.5)	3 (8.6) 20 (57.1) 12 (34.3)	0.051^***^	0.840
Patient’s Relationship				
First-degree relatives Second-degree relatives, etc.	16 (51.6) 15 (48.4)	22 (62.9) 13 (37.1)	0.85^*^	0.356

*  The test conducted is the Chi-Square test

** The test conducted is the Fisher’s test

*** The test conducted is the Chi-Square test for trend

**Table 3:** The effect of mindfulness training on anxiety and sleep quality of informal caregivers of cancer

Mean ± SD				Sig				np^2^
**Anxiety**								
Group	Before intervention	Immediately after intervention	1 month after intervention	Time	Group	Time & Group	Duration of care	0.22
Intervention (31)	105.94 ± 11.73	87.87 ± 16.07	80.39 ± 13.68	< 0.001	< 0.001	< 0.001	0.131	
Control (35)	102.86 ± 16.75	104.23 ± 17.25	112.49 ± 15.91					
**Sleep quality**								
Intervention (31)	11.26 ± 2.65	11.03 ± 2.02	9.94 ± 2.09	0.524	0.004	< 0.001	0.895	0.12
Control (35)	11.8 ± 2.93	12.03 ± 3.03	13.74 ± 2.78					

np^2^ = Partial Eta Squared

## Discussion

This study aimed to assess the impact of mindfulness training on anxiety and sleep quality among informal caregivers of cancer patients. The results revealed a statistically significant difference in pretest and follow-up scores for anxiety and sleep quality between the intervention and control groups.

The findings support the mechanistic role of mindfulness in reducing anxiety, likely through increasing metacognitive awareness, promoting acceptance of negative experiences, and enhancing cognitive flexibility [15, 31, 32]. Specifically, mindfulness may reduce anxiety by modulating neural circuits involved in emotion regulation and fostering a non-defensive orientation to distress. This is consistent with the sustained anxiety reduction observed in the intervention group relative to the control group. In contrast, anxiety scores increased in the control group, consistent with studies by Hasio et al. (2025), Juberg et al. (2023), and Hecht et al. (2021) [17, 18, 33]. The inconsistencies with Kubo et al. (2019) [20] and Bajaj et al. (2017) [21] may stem from differences in intervention modality (mobile app vs. in-person), baseline anxiety levels (floor effect), and cultural context, underscoring the importance of tailoring interventions to specific populations and settings. Unlike Kubo et al., our study included only female caregivers, which may partly explain the different pattern of results, as men often experience distinct emotional and financial caregiving burdens.

The in-person delivery of mindfulness training in this study may have enhanced emotional connection and mutual support through group activities, which could further facilitate anxiety reduction [34]. Caregivers’ continuous exposure to their loved ones’ suffering may heighten existential awareness, which mindfulness, with its emphasis on present-moment acceptance, can effectively address [17].

Given the well-established bi-directional relationship between anxiety and sleep quality, the observed reduction in anxiety likely contributed to improvements in sleep quality. Mindfulness also directly improves sleep by reducing hyperarousal, regulating emotional reactivity, and strengthening attention control mechanisms [13].

The PSQI-based assessment captured the cumulative effects of the intervention, which became significant one-month post-intervention, suggesting that mindfulness training requires sustained practice to yield observable benefits in sleep quality. Our results align with prior studies [17, 21, 35], but differ from Cheung et al. (2020) [36] and Kubo et al. (2019) [20]. These differences may relate to variations in intervention type (acupressure vs. mindfulness), caregiver populations (elderly vs. cancer caregivers), outcome measures (PSQI vs. PROMIS), and timing of assessment (posttest only vs. follow-up), highlighting the need for careful consideration of study design in future research. Again, the fact that our sample was exclusively female is an important factor that may limit comparability with studies including both genders.

Cheung et al.’s population experienced greater physical caregiving burden, whereas cancer caregiving involves profound psychological distress, which may explain differential responsiveness to interventions [35, 36]. Moreover, our younger participant age (mean 39.81 vs. 57.1) may have conferred greater sleep resilience, improving responsiveness to the mindfulness intervention [37]. It should also be noted that unlike some prior studies, our design used a passive control group, which does not fully control for the non-specific benefits of group support or facilitator attention. Kubo’s inclusion of both genders may have also influenced results, as men often bear dual burdens, including financial stress, potentially affecting intervention effectiveness [38].

Mindfulness-based interventions increase cognitive flexibility, enabling caregivers to manage intrusive thoughts and improve sleep patterns [39]. Despite improvements, persistently poor sleep quality suggests that broader structural and contextual factors, such as housing conditions, caregiving intensity, and cultural norms, may limit the effectiveness of mindfulness alone. Therefore, mindfulness should be integrated with additional support strategies to comprehensively address caregiver well-being.

We recommend developing comprehensive caregiver support programs that incorporate mindfulness-based stress reduction alongside psychoeducation, peer support, and practical caregiving skills. Given their close interaction with caregivers, nursing professionals should be trained to deliver these interventions in a culturally sensitive and emotionally supportive manner. Ethical considerations—such as the risk of emotional distress—and logistical challenges, including facilitator preparation and scheduling flexibility, must be addressed, particularly in resource-limited settings.

In line with the holistic nursing perspective, we emphasize the interconnectedness of caregiver and patient well-being. Disruptions in caregivers’ health may compromise patient outcomes, reinforcing the need for caregiver-centered policies in cancer care settings [32].

Although the intervention improved sleep quality, overall scores remained suboptimal. This underscores the complexity of caregiving stress and the need for mixed-methods research to explore caregivers’ lived experiences and contextualize quantitative outcomes. Future studies should not only evaluate combined interventions and longer-term effects, but also include male caregivers, employ active control conditions, and consider strategies to minimize instructor-related bias.

### Limitations & strength

This study has several strengths. It is among the few randomized controlled trials conducted in Iran focusing specifically on informal caregivers of cancer patients, addressing an important gap in regional research. The intervention followed a standardized Mindfulness-Based Stress Reduction (MBSR) protocol, and validated tools—Spielberger State-Trait Anxiety Inventory (STAI) and Pittsburgh Sleep Quality Index (PSQI)—were used to measure outcomes, adding methodological rigor. Furthermore, an intention-to-treat (ITT) analysis was performed, which included all randomized participants regardless of adherence or dropout, enhancing the validity and reliability of the findings.

However, several limitations should be noted. First, the use of convenience sampling may introduce selection bias, limiting the representativeness and generalizability of the findings. Second, although a control group was included, it was a passive control (no intervention). Consequently, the study demonstrates that MBSR is superior to no intervention but does not clarify whether the observed benefits are attributable to specific mindfulness mechanisms or to non-specific factors, such as social support and facilitator attention. Third, blinding was incomplete; neither participants nor facilitators were blinded due to the nature of the intervention, which may have influenced the outcomes. Moreover, the dual role of the instructor—both delivering the intervention and monitoring participants’ adherence (homework and follow-ups)—may have introduced performance bias, a point that should be considered when interpreting the results. Nevertheless, blinding was implemented at the data analysis stage to mitigate bias.

Gender representation was another major limitation. Due to cultural and religious constraints, only female caregivers participated, as male caregivers could not be included without a male instructor. This restriction substantially limits generalizability, since male caregivers may experience different social and emotional burdens. The findings therefore apply only to female caregivers. Future research should consider including both genders, for example by employing instructors of both genders or using remote/online delivery methods to overcome such barriers.

Additionally, the follow-up period was relatively short (one month), which restricts assessment of the sustainability and long-term effects of the intervention. Future studies should include longer follow-up durations to evaluate lasting benefits.

Outcome assessment relied on self-reported questionnaires, which may be subject to social desirability and recall biases. Incorporating objective measures such as actigraphy or physiological assessments is recommended for future research to provide more robust evaluation.

Finally, the cultural specificity of the sample may limit the applicability of the findings to other sociocultural contexts or caregiver populations. Replication in diverse settings and with caregivers of patients with various chronic conditions is needed to strengthen external validity.

In summary, while this study provides preliminary evidence supporting the feasibility and potential benefits of MBSR for informal female caregivers of cancer patients in Iran, the findings should be interpreted with caution in light of the aforementioned limitations. Addressing these issues in future research will be essential to confirm and extend the current findings.

## Conclusion

This study provides preliminary evidence that mindfulness-based stress reduction (MBSR) may help reduce anxiety and improve sleep quality in female informal caregivers of cancer patients. Anxiety reduction was more pronounced, while improvements in sleep quality were moderate but consistent over the follow-up period. These findings indicate that mindfulness interventions could be a useful component of comprehensive caregiver support.

Clinically, incorporating MBSR into caregiver education and support services may offer a practical approach to alleviate psychological distress and sleep problems. Healthcare providers, including nurses and mental health professionals, could consider mindfulness training as part of holistic caregiving support to enhance caregiver resilience. Policymakers are also encouraged to support the inclusion of such interventions in national caregiver support frameworks, particularly in countries with limited psychosocial resources.

However, the findings of this trial should be interpreted with caution in light of several limitations, including the female-only sample (which restricts generalizability to male caregivers who may experience different social and emotional burdens), the short follow-up period, reliance on self-reported measures, the use of convenience sampling, the passive control condition, and the dual role of the instructor in both delivering the intervention and monitoring adherence and the use of a relatively small effect size (f = 0.15) in the sample size calculation. Although this value is commonly accepted in pilot or feasibility studies, it may limit the statistical power to detect clinically meaningful differences in a definitive RCT setting. Future research is warranted to assess the long-term sustainability of effects, incorporate objective outcome measures, and examine the applicability of mindfulness-based interventions across both genders, more diverse caregiver populations, and in comparison, with active control conditions.

## Supplementary Information

Below is the link to the electronic supplementary material.

Supplementary Material 1: Comparison of quantitative baseline characteristics of patients and caregivers between intervention and control groups. This table includes demographic and clinical variables such as Patient’s age, Caregiver’s age, Caring time, Duration of illness and Duration of care, with corresponding statistical comparisons.

Supplementary Material 2: Comparison of qualitative baseline characteristics of patients between the intervention and control groups. This table includes patient’s demographic and clinical variables such as gender, education, presence of another caregiver and cancer type, with corresponding statistical comparisons.

## Data Availability

The data supporting the findings of this study are available from the corresponding author on reasonable request.

## Abbreviations

MBSR: Mindfulness based stress reduction
PSQI: Pittsburgh Sleep Quality Index
STAI: The State-Trait Anxiety Inventory
