# Supplementary material for: The effect of mindfulness-based stress reduction on anxiety and sleep quality in informal family caregivers of cancer patients: a randomized controlled trial
Source: BMC Nurs. 2025 Nov 6;24:1375. doi: 10.1186/s12912-025-04063-z (PMC12593857; doi:10.1186/s12912-025-04063-z)
Supplement: Supplementary file 1 — Supplementary Material 1: Comparison of quantitative baseline characteristics of patients and caregivers between intervention and control groups. This table includes demographic and clinical variables such as Patient’s age, Caregiver’s age, Caring time, Duration of illness and Duration of care, with corresponding statistical comparisons. [file 12912_2025_4063_MOESM1_ESM.docx]

**Additional file 1. Comparison of quantitative baseline characteristics of patients and caregivers between intervention and control groups**

| P value | Mean(SD) | | Variable |
| --- | --- | --- | --- |
|  | **control** | **Intervention** |  |
| 0.842^a^ | 54.20(14.29) | 54.87(12.80) | Patient's age |
| 0.378^a^ | 42.09(10.91) | 39.81(9.82) | Caregiver's age |
| 0.416^b^ | 11.77(9.53)* | 8.35(7.38)* | Daycare hours |
| 0.021^b^ | 1.83(2.70)* | 2.12(1.76)* | Duration of infection (years) |
| 0.025^b^ | 1.83(2.70)* | 2.09(1.76)* | Duration of care (years) |

* Median (interquartile range)

^a^ T test was done.

^b^ Mann Whitney test was done.
