# Supplementary material for: The effect of mindfulness-based stress reduction on anxiety and sleep quality in informal family caregivers of cancer patients: a randomized controlled trial
Source: BMC Nurs. 2025 Nov 6;24:1375. doi: 10.1186/s12912-025-04063-z (PMC12593857; doi:10.1186/s12912-025-04063-z)
Supplement: Supplementary file 2 — Supplementary Material 2: Comparison of qualitative baseline characteristics of patients between the intervention and control groups. This table includes patient’s demographic and clinical variables such as gender, education, presence of another caregiver and cancer type, with corresponding statistical comparisons. [file 12912_2025_4063_MOESM2_ESM.docx]

| **P value** | **Fisher or x2** | **Control group frequency (%)** | **Intervention group frequency (%)** | **Variable** |
| --- | --- | --- | --- | --- |
| 0.244 | 1.35^*^ | 20 (57.1)  15 (42.9) | 22 (71)  9 (29) | Gender  Female  Male |
| 0.510 | 2.52^**^ | 20 (57.1)  11 (31.4)  2 (5.7)  2 (5.7) | 16 (51.6)  11 (35.5)  4 (12.9)  0 (0) | Education  under diploma  diploma  Bachelor  Master |
| 0.148 | 2.09^*^ | 24 (68.6)  11 (31.4) | 26 (83.9)  5 (16.1) | Presence of another caregiver  Yes  No |
| 0.163 | 3.62^*^ | 8 (22.9)  10 (28.6)  17 (48.6) | 10 (32.3)  13 (41.9)  8 (25.8) | Cancer type  Abdomen  Breast  Other |

**Additional file 2. Comparison of qualitative baseline characteristics of patients between the intervention and control groups**

* The test conducted is the Chi-Square test.

** The test conducted is the Fisher's test.
